# Does girls’ empowerment predict contraceptive intentions? Evidence from a survey of secondary school girls in Northwest Nigeria

**DOI:** 10.1080/26410397.2022.2146034

**Published:** 2023-03-06

**Authors:** Angubeen G. Khan, Paula Tavrow, Fatima Adamu

**Affiliations:** a PhD Student, UCLA Fielding School of Public Health, Los Angeles, CA, USA*Correspondence*: angukhan@ucla.edu; b Adjunct Professor, UCLA Fielding School of Public Health, Los Angeles, CA, USA; c Executive Director, Nana Girls and Women Empowerment Initiative, Sokoto, Nigeria

**Keywords:** adolescence, family planning, intentions, Nigeria, empowerment

## Abstract

In sub-Saharan Africa, women’s empowerment has been linked to contraceptive use, but little is known about whether girls’ empowerment affects contraceptive intentions, particularly in more traditional societies where early marriage and childbearing are common. Drawing on a survey of 240 secondary school students in Kebbi State, Northwest Nigeria, in September–November 2018, we examined whether dimensions of girls’ empowerment (academic self-mastery, perceived career feasibility, progressive gender norms, and marriage autonomy) and family planning indicators (knowledge, desired family size) were associated with future intentions to use family planning. We found that half of the girls had no intention to use contraception, and only one-fourth intended to use contraception for both delaying/spacing and stopping pregnancies. Multivariate analysis revealed that one dimension of empowerment (perceived career feasibility) and family planning knowledge were significantly associated with intentions. These results suggest that girls perceive contraceptive use as risky, and require contraceptive knowledge and an anticipated career to overcome their trepidation. To increase girls’ intentions to use contraceptives, it is vital that they receive comprehensive sexuality education and career counselling.

## Résumé

En Afrique subsaharienne, l’autonomisation des femmes a été liée à l’utilisation de contraceptifs, mais on ignore si l’autonomisation des jeunes filles influe sur leurs intentions en matière de contraception, en particulier dans les sociétés plus traditionnelles où le mariage et la maternité à un âge précoce sont fréquents. Se fondant sur une enquête auprès de 240 élèves de l’enseignement secondaire dans l’État de Kebbi, au nord-ouest du Nigéria, en septembre-novembre 2018, nous avons étudié si les dimensions de l’autonomisation des filles (maîtrise scolaire, faisabilité perçue du projet professionnel, normes de genre progressistes et autonomie en matière de mariage) et les indicateurs de planification familiale (connaissance, nombre d’enfants souhaité) étaient associés aux intentions futures d’utiliser la planification familiale. Nous avons observé que la moitié des jeunes filles n’avaient pas l’intention d’utiliser la contraception, et qu’à peine un quart envisageaient d’avoir recours à la contraception pour retarder/espacer et éviter des grossesses. Une analyse multivariée a révélé qu’une dimension de l’autonomisation (faisabilité perçue du projet professionnel) et la connaissance de la planification familiale étaient associées de manière significative avec les intentions. Ces résultats semblent indiquer que les filles jugent que l’emploi de contraceptifs est risqué et qu’elles ont besoin d’une connaissance des contraceptifs et d’un projet professionnel pour surmonter leur appréhension. Pour augmenter l’intention des filles d’utiliser des contraceptifs, il est essentiel de leur dispenser une éducation sexuelle complète et des conseils sur leur projet professionnel.

## Resumen

En África subsahariana, el empoderamiento de las mujeres ha sido asociado con el uso de métodos anticonceptivos, pero no se sabe mucho sobre si el empoderamiento de las niñas afecta las intenciones anticonceptivas, en particular en sociedades más tradicionales donde el matrimonio y la maternidad precoces son comunes. A raíz de una encuesta de 240 estudiantes de escuela secundaria en el estado de Kebbi, en el noroeste de Nigeria, entre septiembre y noviembre de 2018, examinamos si las dimensiones del empoderamiento de las niñas (autodominio académico, factibilidad de carrera percibida, normas de género progresistas y autonomía matrimonial) y los indicadores de planificación familiar (conocimiento, tamaño de familia deseado) estaban asociados con futuras intenciones de usar planificación familiar. Encontramos que la mitad de las niñas no tenía ninguna intención de usar anticoncepción y solo una cuarta parte tenía la intención de usar anticoncepción para retrasar/espaciar y evitar embarazos. El análisis multivariable reveló que una dimensión del empoderamiento (la factibilidad de carrera percibida) y el conocimiento de planificación familiar estaban asociados significativamente con las intenciones. Estos resultados indican que las niñas perciben el uso de anticonceptivos como riesgoso y que necesitan conocimiento de anticonceptivos y una carrera profesional prevista para superar su temor. Para aumentar las intenciones de las niñas de usar anticonceptivos, es vital que ellas reciban educación integral en sexualidad y orientación profesional.

## Introduction

Efforts to encourage more contraceptive use in traditional Muslim societies usually begin with assessments of married women’s unmet need for family planning. Included in this assessment is asking fertile women who are not currently using a contraceptive method whether they intend to use one in the future. Studies suggest that married women’s stated contraceptive intentions in surveys are predictive of future contraceptive use if they have access to contraceptives and were not overly influenced by an interviewer.^1^ While goal intentions (what people plan broadly for their future) have been found to be less predictive of behaviour than implementation intentions (what specific actions people plan to take in a certain time horizon), goal intentions are still considered an important step on the path to later contraceptive use.^2,3^ Using modern contraception to delay, space or stop childbearing requires intentionality, forethought and actions from one or both partners. If a societal goal is to avoid unintended pregnancies and delay childbearing after marriage, contraceptive use ideally commences prior to or shortly after sexual debut.

Given that it is a standard practice to ask non-users of contraception about their future intentions, it is curious that unmarried adolescent girls are rarely asked similar questions. In reviewing the literature from lower income countries, we found numerous instances where unmarried adolescents were asked about their current sexual activity and contraceptive use, (e.g.^4,5^) and a few about their fertility intentions.^6,7^ However, we were unable to identify any studies where girls who were not sexually active were asked about their future intentions to use contraception. We believe this represents a major gap in our knowledge base. Estimating the extent of adolescents’ willingness to use contraception in the future, and determining what factors might account for their intentions, would enable government agencies and non-governmental organisations to develop more appropriate interventions to avoid early or unwanted pregnancies among young or newly married women.

This is particularly important in traditional Muslim regions such as Northwest Nigeria, where premarital sexual intercourse among Hausa girls is low, the median age of marriage is 16, and the median age of first birth is 17.6.^8,9^ Many Hausa girls are physically, emotionally, and financially unprepared for motherhood at the time of their first birth.^10^ They are vulnerable to numerous adverse consequences, including maternal mortality and morbidity, preterm birth and neonatal death, as well as lost educational and economic opportunities.^11^ Hausa women in Nigeria have an average fertility rate of 6.5, among the highest in Africa, as well as an estimated unmet need for family planning among married women of 10–15%.^9^

Previous studies from sub-Saharan Africa have found that various components of women’s empowerment are positively associated with contraceptive use, such as decision-making autonomy, spousal communication, participation in the labour force, and knowledge about contraceptive methods.^12^ However, these measures are often developed for adult women and include items such as autonomy in the household and decision-making vis-à-vis their spouse. Very few studies have measured girls’ empowerment, and none have examined possible links to contraceptive intentions or use. A recent review of measurement methods for women’s and girls’ empowerment conducted by the Bill and Melinda Gates Foundation^13^ identified only two instruments that specifically measured girls’ empowerment, of which one was a qualitative checklist for health staff and the other a non-validated survey instrument that did not include contraceptive intentions.^14^ Two studies published after the Gates review have examined the effects of African girls’ empowerment on health, but neither examined intentions to use contraception, and the girls’ empowerment measures used were closely linked to project goals of anti-smoking^15^ or asset-building.^16^ Clearly, there is a need for girls’ empowerment measures that are tailored to specific populations, such as traditional Muslim societies. These measures could be used to assess the role of girls’ empowerment in those societies on various health issues, such as contraceptive intentions and use.

The objectives of this paper were twofold. The first was to develop a new index of empowerment for girls in traditional societies. The second was to assess whether this new index, or individual elements of the index, were associated with girls’ intentions to use contraception after marriage. Our goal was to provide insights into factors that could shape girls’ future contraception decisions in these societies.

## Materials and method

### Sample

The data used for this study were drawn from a baseline survey of unmarried girls ages 13–19 years who were in the third cohort of the Girls for Health (G4H) programme in Kebbi State. This programme, funded by the Bill & Melinda Gates Foundation, was designed to assist rural adolescent girls in their final two years of secondary school to enter health-related careers such as medicine, midwifery, and nursing, to address the shortage of female health providers in northern Nigeria. Because gender norms in this region mandate that women receive reproductive care only from other women, having insufficient female health providers means that some women do not receive any skilled health care during pregnancy and delivery. G4H’s goal was to help reduce this shortage by improving secondary school girls’ core academic skills in science, mathematics and English, so that they could qualify for tertiary education in various health fields.

### Data collection

The baseline survey was conducted from September to November 2018 with girls in their last two years of instruction at five rural high schools (senior level of secondary schools), both day and boarding schools. Interviewers were four men and three women drawn from the staff of G4H and an associated programme. Interviewers received three days of training on the survey instrument and procedures. Each survey was conducted in Hausa, took about 90–100 minutes to administer, and occurred in a private office on the school grounds, to ensure that the girls were not overheard by others. Girls were not queried directly about their prior sexual activity and contraceptive use, although they were asked if anyone had ever tried to rape them, to which four responded in the affirmative. The goal was to interview 250 girls participating in G4H (about 50 from each school).

Ethical approval for G4H was granted by Federal University’s Research Ethical Committee, Kebbi State, and the University of California at San Diego’s Institutional Review Board (IRB#160417S) in July 2016. Prior to the start of the G4H program, the project team met with parents and guardians to discuss all aspects of the programme including the baseline survey. Parents and guardians orally gave informed consent for girls to participate in the programme and survey. Before the administration of the survey, girls gave their voluntary informed consent (or assent in the case of minors) to participating in the baseline survey. Girls were assured that their responses would be kept confidential.

### Measures

#### Dependent variable

Intention to use contraception was measured using two items from the baseline survey that asked whether respondents expected to use a contraceptive method once married. The first question asked about intended contraceptive use for spacing pregnancies, and the other about use for stopping childbearing after reaching desired family size. A single dichotomous variable was created using these items with the following categories: no intention to use contraception for spacing or stopping once married (0); intend to use for spacing/delaying and/or stopping childbearing (1). We categorised “don’t know” as equivalent to “no intention”.

#### Independent variables

Because existing empowerment measures were developed for adult women and included items such as autonomy in the household and decision-making vis-à-vis their spouse, we created new indices of girls’ empowerment that were relevant for traditional societies where female agency is highly constrained, especially during adolescence. In developing our indices, we drew from Karp et al.’s^17^ conceptual model for sexual and reproductive health empowerment of women and girls in sub-Saharan Africa. This framework describes societal and individual level factors that influence the achievement of autonomy and choice for girls and women across their life course. For adolescents, *existence of choice* – defined as girls’ capacity to realise their reproductive goals – is a key component of empowerment.^17^ Being able to make strategic life choices is affected by *external* forces, such as power relations with family members and broader community norms about women’s roles, and by *internal* factors, such as individual self-esteem and achievement.^18,19^

**Figure 1.**
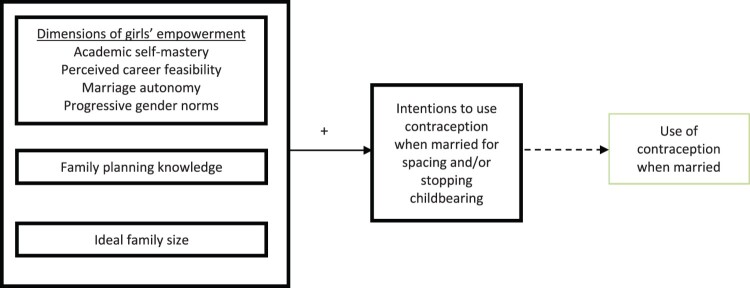
Conceptual model of future contraceptive intentions of secondary school girls in Kebbi State, Nigeria

Drawing on the Karp et al.^17^ empowerment framework for sub-Saharan Africa, we aimed to develop girls’ empowerment measures to operationalise the concept of *existence of choice* that would encapsulate external factors (power relations with guardians and gender norms) and internal factors (girls’ self-esteem and achievements). Using questions from the G4H baseline survey of adolescent girls in Northern Nigeria, we created four indices which represented four dimensions of girls’ empowerment in this setting, including academic self-mastery and perceived career feasibility (represented internal forces), and progressive gender norms and marriage autonomy (represented external forces) (Figure 1).

*Academic self-mastery* was defined as how well a girl believes she can handle her coursework and used to approximate self-esteem and achievement. To create the index for *academic self-mastery,* we used four survey items: whether respondents felt confident that they could master skills taught in school, could do well if they had enough time for their classwork, could learn difficult concepts, and could learn new concepts quickly. Respondents had four response choices which we dichotomised as follows: “agree fully” indicated the highest level of academic self-mastery (1); and “agree some”, “disagree”, and “don’t know” indicated lower academic self-mastery (0). A score of four indicated that the respondent felt she could handle all of her coursework.

*Perceived career feasibility* represented whether girls believed that their families would allow them to pursue tertiary education and careers. For the *perceived career feasibility* index, we used five survey items that assessed if respondents could get extra time to devote to their studies if needed; whether their parents would be willing, could afford, or would allow them to attend tertiary school; and if they had a say on whether they would attend tertiary school in the future. Response choices for each item were: “yes, always”, “sometimes”, “no” and “don’t know”. All items were dichotomised where “yes, always” indicated the highest level of perceived career feasibility (1) and “sometimes”, “no”, and “don’t know” indicated lower perceived career feasibility (0). A score of five indicated that the respondent felt it was very likely she could pursue a career.

*Progressive gender norms* were defined as beliefs about gender equality and created to capture community norms. For the *progressive gender norms* index, we relied on five attitudinal items from the baseline survey that assessed traditional gender norms. These items included three questions regarding prioritisation of education of sons over daughters, one question about whether women should leave politics to men, and one question about whether a good woman should never question her husband’s opinion. We dichotomised response choices for each of these items as “agree” and “don’t know” (1), and “disagree” (0). A score of 0 indicated the respondent had more progressive gender norms, but we reversed it so that a score of five was the most progressive.

To address family dynamics, we created the index for *marriage autonom*y which was defined as whether girls will have a role in choosing when and whom to marry. To create the *marriage autonomy* index, we chose four survey items concerning whether the respondent would be able to choose when to marry. These items included three questions on whether respondents felt pressure from or could communicate with their parents/guardians on the timing of marriage, and one item on if they could stop someone in their household from pressuring them to get married. We dichotomised the responses so that no pressure and confidence in one’s ability to communicate concerns or stop marriage pressure indicated high marriage autonomy (1), while greater pressure to marry, inability to communicate concerns or stop marriage indicated lower marriage autonomy (0). A score of four indicated that the respondent felt agentic in deciding her age of marriage.

After selecting items from the survey that represented each of the indices, we performed factor analysis to confirm that the items chosen for each index had an acceptable Cronbach’s alpha (>0.60). The indices were all on a continuous scale. Higher index values represented greater empowerment for marriage autonomy, academic self-mastery, and perceived career feasibility. For the progressive gender norms index, lower scores represented more progressive gender norms, which we then reversed to align with the other indices.

Because knowledge of family planning methods and lower desired family size have also been linked to contraceptive use and birth spacing among adult women,^20–23^ we also included these survey items in our predictors of girls’ intentions to use contraception. For this study, family planning knowledge was measured using one survey item of whether respondents specifically knew of any methods to delay or avoid pregnancy, in which we assigned “yes” (1) and “no” or “don’t know” (0). Ideal family size was measured using a survey question on how many children the respondent wished to have in the future, which in this survey had only three response choices: “1–5 children”, “6–10 children,” and “don’t know”. We dichotomised these response choices as: “1–5 children” (1), and “6–10 children” (0). Only eight respondents (3.3%) reported that they did not know their ideal family size. We placed these respondents into the category of “6–10 children” because we construed ambivalence as being inclined towards a traditional larger family size.

#### Control variables

In our analysis we controlled for the respondent’s age, mother’s ability to read English, and poverty. Age is known to influence reproductive intentions in adult women,^24^ but less is known about adolescents.^25^ We chose to control for any variation due to age that may occur in the sample, since the respondents’ ages spanned from 13 to 19, even though all were in their final two years of secondary school. Because socialisation theories suggest that parents have a strong role in influencing young adult behaviours,^26^ we also controlled for mother’s empowerment by including mother’s education in the analysis. Past studies indicate that when mothers have higher status, such as higher level of education, their daughters are more likely to delay sexual initiation.^27^ Finally, we controlled for girls’ socioeconomic status by creating an index of poverty using two items from the survey: if the wall material of the respondent’s home was mud and if the respondent ever had to skip a meal in the last three months because she could not afford it. A higher score (2) indicated lower socioeconomic status.

#### Analysis

We first conducted univariate analysis of the demographic characteristics, family planning knowledge, desired family size, and intentions to use contraception. Then we created four indices of girls’ empowerment, calculated Cronbach alphas of each index to assess their reliability, and obtained descriptive statistics including the mean, standard deviation, and range of each index. Next, we performed a binomial logistic regression to examine factors that were associated with future intentions to use contraception. We also conducted sensitivity analyses to confirm that results did not significantly change when “don’t know” responses were combined with “no” for the dependent variable (not shown). All analyses were performed in SPSS version 27.

## Results

### Descriptive results

The analytic sample from the baseline survey consisted of 240 adolescent girls between the ages of 13–19, of whom 62% were 16–17 (see Table 1). About half of the girls were in the first year of senior secondary school, and the remainder were in their second (final) year. Though the girls participating in the programme were selected from among their peers based on their rural residence and high performance in public schools, less than 40% could read a full sentence in English. Nearly three-fourths of the girls came from large families with six or more siblings. About 20% had at least one deceased parent. Few girls had mothers with a professional career (13%) or who could read English (43%), although 39% had a father with a professional vocation and 66% had a father who could read English. As indicators of poverty, about 43% of the girls had skipped lunch in the past three months because they could not afford it, and 50% lived in a house with mud walls.

**Table 1.** Demographic characteristics of girls in secondary school in Kebbi State, Nigeria (*N* = 240)

	*N*	%
*Age*		
3–15	50	(20.8)
16–17	149	(62.1)
18–19	41	(17.1)
*Senior secondary school level*		
First year	116	(48.3)
Second year	124	(51.7)
*Respondent’s ability to read English from a card*		
Able to read whole sentence	92	(38.3)
Able to read only part of the sentence	128	(53.3)
Cannot read at all	14	(5.8)
Missing	6	(2.5)
*Number of siblings*		
0	1	(0.4)
1–5	67	(27.9)
6–10	115	(47.9)
11+	57	(23.8)
*Has living parent(s)*		
Both parents are deceased	5	(2.1)
Father only is deceased	31	(12.9)
Mother only is deceased	11	(4.6)
Both parents are alive	193	(80.4)
*Mother’s livelihood*		
Professional	31	(12.9)
Selling and farming	114	(47.5)
Unemployed or homemakers	73	(30.4)
Deceased or unspecified	22	(9.2)
*Mother is able to read English*	104	(43.3)
*Father’s livelihood*		
Professional	93	(38.8)
Selling and farming	91	(37.9)
Unemployed/retired	10	(4.2)
Deceased or unspecified	46	(19.2)
*Father is able to read English*	159	(66.3)
*Poverty*		
Had to skip lunch at least once in past 3 months	102	(42.5)
*House material is mud*	120	(50.0)

Knowledge of methods to prevent pregnancy, ideal family size, and intentions to use contraception in the future are shown in Table 2. Family planning knowledge was low, with only 27% reporting they knew specific methods to prevent or avoid pregnancy. Most (71%) wanted a family of five or fewer children, yet nearly half of the sample had no intention to use contraception in the future or didn’t know. Approximately half (*N* = 123) of the girls intended to use contraception in the future to either space/delay future pregnancies and/or to stop childbearing.

**Table 2.** Contraceptive knowledge, desires and intentions among girls in secondary school in Kebbi State, Nigeria (*N* = 240)

	*N*	%
*Knowledge of methods to prevent or avoid pregnancy*		
Yes	64	(26.7)
No	176	(73.3)
*Ideal family size*		
1–5 children	171	(71.3)
6–10 children	61	(25.4)
Undecided	8	(3.3)
*Intention to use contraception once married*		
Does not intend to use contraception at all^a^	117	(48.8)
Intend to use for spacing, delaying and/or stopping childbearing	123	(51.3)

^a^ Includes 13 girls who responded “don’t know”.

### Reliability analysis

In Table 3, we report the results of the reliability analysis we conducted to form four new measures of girls’ empowerment. In comparing the four indices, we found that girls reported significantly more academic self-mastery and perceived career feasibility than progressive gender norms and marriage autonomy. The girls in this sample had very high levels of academic self-mastery, with 70–82% reporting confidence in their ability to do well in school. Most girls in this programme also had high levels of perceived career feasibility, with 83% believing that their parents would want them to attend a tertiary educational institution if they passed their exams. However, because of poverty, only 66% believed that their parents could afford to send them to a tertiary institution. Concerning gender norms, only 28% of girls thought that sons should have more education than daughters, but more than two-thirds subscribed to traditional norms about women’s proper place in the home and in politics. Regarding marriage autonomy, the vast majority (83%) of the sample reported that they did not feel pressure to marry soon, but only two-thirds felt comfortable discussing with their parents when they would marry and just 52% felt that they could stop the marriage.

**Table 3.** Reliability of dimensions of empowerment among girls in secondary school in Kebbi State, Nigeria

Index	Items	%
**Academic self-mastery**	1. If I have enough time, I can do a good job on all my class work.	81.7
Alpha = 0.89	2. I am confident that I can master the skills taught in school this year.	75.8
Mean = 3.78	3. Even if the work is hard, I can learn it.	74.2
SD = 1.79	4. I learn new concepts quickly.	69.6
**Perceived career feasibility**	1. If I pass my exams, my parents or guardian will want me to go to a tertiary institution.	83.3
Alpha = 0.82	2. I have a say in my household on whether I attend a tertiary institution.	78.3
Mean = 4.43	3. If I am accepted to a tertiary institution that is far from here, my family will allow me to move there.	71.7
SD = 1.83	4. If I pass my exams, my parents or guardian will be able to afford to send me to a tertiary institution.	66.3
	5. If I need more time to devote to my studies, I am always able to get it.	64.6
**Progressive gender norms**^a^	1. Women should leave politics to men.	67.5
Alpha = 0.68	2. A good woman never questions her husband’s opinions, even if she is not sure she agrees with them.	67.1
Mean = 2.38	3. If there is a limited amount of money to pay for tutoring it should be spent on sons first.	51.7
SD = 1.58	4. The most important reason that sons should be more educated than daughters is so that they can better look after their parents when they are older.	47.5
	5. It is important that sons have more education than daughters.	28.3
**Marriage autonomy**	1. I do not feel pressure from my family to get married soon after I finish your secondary school.	83.3
Alpha = 0.61	2. My parent or guardian would support me if I wanted to delay marriage.	76.7
Mean = 2.79	3. I can talk easily to my parents or my guardians about when and what age I would like to get married.	66.7
SD = 1.21	4. If someone in my household tries to pressure me to get married, I can find someone to stop it, if I like.	52.1

^a^ Prevalence of “agree” and “don’t know” responses are reported. The items on the index were reverse coded to represent “progressive gender norms” in the subsequent binomial logistic regression analysis.

In assessing the reliability of the indices, academic self-mastery and perceived career feasibility had the highest levels of reliability, as indicated by Cronbach alpha scores of 0.89 and 0.82, respectively (see Table 3). The indices for gender norms and marriage autonomy had considerably lower Cronbach alpha scores, 0.68 and 0.61, respectively, but these are still considered acceptable levels. Removing items from these indices would not raise the alpha scores.

### Regression results

In Table 4, we present two models. The first model shows the unadjusted odds ratios of intention to use contraception after marriage, regressed on various dimensions of girls’ empowerment and family planning indicators. After adjusting for controls, in the second model we found that perceived career feasibility was significantly associated with intentions to use contraception in the future. That is, for each unit increase in girls’ levels of confidence in their ability to pursue tertiary education after secondary school, girls were nearly 50% more likely to intend to use a method of contraception after marriage for delaying/spacing and/or stopping childbearing (*p* = 0.003). Of the family planning indicators, specific knowledge of methods to prevent or delay pregnancy was also a significant predictor of intentions to use contraception after marriage (*p* = 0.004). To test if the gender of the interviewer made a difference, we conducted a sensitivity analysis and found no effect (results not reported).

**Table 4.** Binomial logistic regression of dimensions of empowerment on intention to use contraception after marriage among girls in secondary school in Kebbi State, Nigeria (*N* = 240).

	O.R. (s.e.)	95% Confidence Interval		O.R. (s.e.)	95% Confidence Interval	
**Dimensions of girls’ empowerment**						
Academic self-mastery	0.962	0.79	1.172	1.003	0.819	1.23
	(0.101)	–	–	(0.104)	–	–
Perceived career feasibility	1.503*	1.175	1.921	1.467*	1.142	1.884
	(0.125)	–	–	(0.128)	–	–
Gender norms	1.004	0.836	1.207	0.997	0.829	1.2
	(0.094)	–	–	(0.094)	–	–
Marriage autonomy	1.254	0.911	1.727	1.280	0.925	1.773
	(0.163)	–	–	(0.166)	–	–
**Indicators of family planning**						
Knowledge of methods to prevent or avoid pregnancy	2.425*	1.26	4.67	2.692*	1.365	5.312
	(0.334)	–	–	(0.347)	–	–
Ideal family size (ref. 1–5)	1.270	0.668	2.418	1.132	0.582	2.203
	(0.328)	–	–	(0.340)	–	–
**Controls**						
Age of respondent	–	–	–	1.180	0.727	1.915
	–	–	–	(0.247)	–	–
Mother can read English	–	–	–	0.945	0.525	1.702
	–	–	–	(0.300)	–	–
Poverty index	–	–	–	1.314	0.916	1.884
	–	–	–	(0.184)	–	–
Pseudo *R*^2^	0.220			0.237		

Notes: (1) We report the Nagelkerke Pseudo *R*^2^; (2) standard errors are in parentheses; (3) *p < 0.01; **p < 0.001.

## Discussion

Women’s empowerment has been found to be an important predictor of contraceptive use in Africa, and increasing their agency and autonomy could be effective strategies for reducing unmet need for contraception.^28^ Past studies show positive associations between contraception use and educational attainment or employment, or women’s having a say in such matters.^12,29^ This study suggests that giving attention to girls’ empowerment, particularly in traditional Muslim societies, could similarly increase contraceptive intentionality, thereby leading to heightened use.

Our research found that for secondary school female students living in a society with highly restrictive gender norms, intentions to use contraceptive methods in the future were very low, with only one-quarter planning to use contraception after marriage for spacing/delaying and stopping. Clearly, adolescents had major misgivings about modern contraceptives. However, our results suggest that one notable aspect of girls’ empowerment – enhancing their abilities to pursue tertiary education and embark on a professional career – could lead to greater contraceptive use. We speculate that perceived career feasibility is associated with contraceptive intentions because young women believe that they could “take a chance” with contraceptives impairing their fertility or causing other harms, if they felt confident that their career would give them financial security and status. A study of middle school students in rural Guatemala had similar findings. Researchers determined that children’s fertility intentions were influenced by parent’s investment in their child’s education and children’s expectations of their own educational and occupational achievements.^6^ While this Guatemala study did not examine contraceptive intentions, it did give credence to the idea that future career expectations can shape adolescent fertility intentions. Moreover, by assisting their children to have an education and career, parents can have an integral role in shaping adolescent family planning intentions.

Another key implication from our study was that providing information about contraceptive methods to female students could increase their agency regarding future contraceptive use. Our findings agree with past studies that report knowledge as a significant determinant of contraceptive usage,^30,31^ although we were focused on girls from traditional societies who had never used contraceptives. Like other studies from the African region, we found that knowledge of specific forms of contraception among adolescents was low, mainly because parents avoid discussing this topic with their children and sexuality education in schools is limited or non-existent.^31–33^

Interestingly, none of the other empowerment dimensions we tested were predictive of intentions to use contraception among the youth in our sample. Our study indicated that progressive gender norms were not associated with future intentions to use contraception. Although there is an extensive literature that indicates that future intention to use contraception is related to several measures of women’s empowerment,^28,34–38^ these studies focused primarily on married women. Studies concerning women with no children or adolescents have mixed results in relation to the role of descriptive norms and contraceptive intentions.^39,40^

We had expected that girls with higher levels of academic self-mastery would be more likely to intend to use contraception in the future. However, the results indicate that doing well in school is not sufficient for girls to feel that they can take the risk of using contraception in the future, probably because high grades in school do not necessarily translate into financial independence or status for girls, who are still under the control of parents. By the same token, being able to decide when to marry does not in itself confer status or give security, even though it might permit young women to begin a career before getting married.

We also expected that preference for smaller family size would be positively associated with willingness to use contraception in the future, but the relationship was not significant. This may in part relate to a deficiency in the responses to the survey question, because the responses were limited to “1–5 children” and “6–10 children”, which failed to capture smaller family size ideals which could be linked to contraceptive intentions. Moreover, albeit such a response would be rare in this society, no one responded that they had no desire for children.

Plotnick^7^ has noted that adolescents make childbearing decisions using a thorough evaluation of the opportunity cost of delaying those experiences.^7^ Likewise, the girls in this sample may have made an opportunity cost calculation regarding future contraceptive use. With little knowledge of methods to prevent pregnancy, many undoubtedly harboured misconceptions prevalent in sub-Saharan Africa that using contraception could lead to female promiscuity, male infidelity, cancer, weakness or infertility.^1,30,31,33,41–44^ Infertility fears are particularly influential since high fertility is valued and infertility has major social costs, such as abandonment by a male partner, husband taking another wife,^45–47^ ridicule, mistreatment, and familial or community condemnation.^45,48,49^ Moreover, girls may worry about being isolated later in life^45,50,51^ or lacking the support of children and facing economic hardship in old age.^52,53^

In sum, our results indicate that when adolescents in traditional societies such as Northwest Nigeria perceive high risks of using contraception based on lack of knowledge and a low likelihood of attaining a professional career, few will intend to use contraceptives for spacing and stopping childbearing in the future. These adolescent intentions likely will result in low uptake of contraceptive methods when these young women marry, which for some could be soon after they complete secondary school. To increase contraception intentionality, it is important that schools offer comprehensive sexuality education that includes accurate information on contraceptives. In addition, health providers could visit schools and community gatherings to demystify contraceptive methods and discuss their safety for young people. Also, mass media edutainment could be valuable if it depicted nulliparous women safely using contraceptives and then having children when they discontinue use.

To enhance perceived career feasibility, it is recommended that high schools employ career counsellors who can assist girls to make career choices, as well as hold workshops for parents to advise them on how to support their daughters to plan for their futures. Furthermore, Nigeria could seek to reduce financial barriers to higher education and to incentivise professional internship opportunities for young women. These types of activities would likely increase young women’s demand for contraception, which would be expected to reduce early and unwanted childbearing.

This study had several limitations. One limitation was that the survey was conducted with the assistance of interviewers. Although the responses were self-reported and confidential, they were not anonymous. There was a risk of social desirability bias if the girls in the study did not feel comfortable disclosing honest responses to questions on potentially sensitive subjects such as contraceptive intentions and knowledge. However, because the interviewers were not school teachers or staff, this limitation was mitigated. The study’s generalisability is also limited since the study participants were preselected for the G4H based on their school performance and rural residence. As such, the study findings are not representative of all adolescent girls in the region. Contraceptive intentions might be even lower for girls not in G4H or similar programmes. Lastly, some limitations related to the survey questions. For family planning knowledge, we could not assess if self-reported knowledge reflected accurate information about contraceptive methods. In addition, as previously noted, the question about ideal family size used only two response categories. Having continuous response choices may have elicited different answers. Notwithstanding these limitations, this study gave new insights into the understudied topic of girls’ empowerment and contraceptive intentions in a traditional society.

## Conclusion

Adolescence is a critical stage in which young people develop agency and transition from the roles and responsibilities of childhood to those of adulthood. Young adults’ trajectories and health outcomes are shaped by community and social norms, socialisation, education, and familial and peer relationships. Given that most adolescent girls in traditional societies marry before the age of 20, identifying the factors that influence their contraceptive intentions may provide valuable information to programme planners who seek to reduce early or unwanted childbearing and enhance female self-actualisation.

In this study, we focused on the future contraceptive intentions of secondary school girls in a traditional Muslim Hausa society in Northern Nigeria. The girls in this study were at a critical juncture in their lives when decisions related to their occupation trajectories, marriage, and family formation, were imminent. Our results suggest that some domains of girls’ empowerment were linked to contraceptive intentions, which in turn are likely to predict their uptake of contraception after marriage. For those wishing to increase young women’s use of contraception and reduce early and unwanted childbearing, a starting point might be to enhance girls’ empowerment by providing them with accurate information about contraceptives and assisting them to realise their career aspirations.
